# Global Landscape of Clinical Studies Open to Enrollment in Carbon Ion Radiotherapy: A PTCOG Review

**DOI:** 10.1016/j.ijpt.2026.101968

**Published:** 2026-10-06

**Authors:** Adam L. Holtzman, Huan Giap, Hitoshi Ishikawa, Tatsuya Ohno, Guoliang Jiang, Qiang Li, Keng-Li Lan, Woong Sub Koom, Sebastian Adeberg, Ester Orlandi, Piero Fossati, Eugen Hug, Jacques Balosso, Semi Harrabi, Bradford S. Hoppe

**Affiliations:** 1 Mayo Clinic, Jacksonville, FL, USA; 2 OSF HealthCare, Princeton, IL, USA; 3 National Institutes for Quantum Science and Technology (QST; Chiba/HIMAC), Chiba, Japan; 4 Gunma University Heavy Ion Medical Center, Maebashi, Japan; 5 Shanghai Proton and Heavy Ion Center (SPHIC), Shanghai, China; 6 Institute of Modern Physics, Chinese Academy of Sciences, Lanzhou Heavy Ion Hospital, Lanzhou, China; 7 Department of Heavy Particles and Radiation Oncology, Taipei Veterans General Hospital, Taipei, Taiwan; 8 Yonsei Cancer Center, Yonsei University Health System, Seoul, South Korea; 9 Department of Radiation Oncology, University Hospital Giessen and Marburg (UKGM), Marburg, Germany; Marburg Ion-Beam Therapy Center (MIT), Marburg, Germany; 10 University Cancer Center (UCT) Frankfurt-Marburg, Marburg and Frankfurt, Germany; 11 Department of Clinical, Surgical, Diagnostic, and Pediatric Sciences, University of Pavia, Pavia, Italy; 12 MedAustron Ion Therapy Center, Wiener Neustadt, Austria; 13 Grenoble Alpes University, Grenoble, France; 14 Heidelberg Ion-Beam Therapy Center (HIT), Heidelberg University Hospital, Heidelberg, Germany; 15 RadiationOncology Unit, Clinical Department, CNAO National Center for OncologicalHadrontherapy, Pavia, Italy

**Keywords:** Carbon ion radiotherapy, Heavy ions, Clinical trials, Particle therapy

## Abstract

**Background:**

Radiotherapy with high linear energy transfer (LET) has shown promise for improving cancer outcomes. Carbon ion radiotherapy (CIRT) combines charged-particle dose conformality with enhanced biological effectiveness. However, clinical development remains constrained by limited center availability, modest trial accrual, and few randomized studies. We reviewed the global landscape of CIRT studies open to enrollment to identify current priorities and gaps.

**Methods:**

This Particle Therapy Cooperative Group (PTCOG)-sanctioned project, conducted within the CIRT Guidelines Subcommittee, identified studies through registry review and institutional verification. ClinicalTrials.gov and the Japan Registry of Clinical Trials were searched from December 2025 through February 2026. We included interventional CIRT trials and prospective registries open to new enrollment and excluded studies not open to enrollment, retrospective cohorts, proton-only studies, and preclinical studies.

**Results:**

Thirty-six studies were identified: 35 interventional trials in 6 countries and 1 North American registry-based comparative-effectiveness study. Japan had the largest share, followed by Germany, China, Italy, Austria, and France. Three centers (SPHIC, QST, and HIT) accounted for 21 of 36 studies. The portfolio included 7 phase I, 23 phase II, 5 phase III, and 1 registry study. Pancreatic cancer and sarcoma-related tumors each accounted for 7 studies; head and neck cancers, 5; prostate and breast cancers, 3 each; glioblastoma, lung cancer, and gynecologic malignancies, 2 each; and mucosal melanoma, mixed solid tumors, kidney cancer, esophageal cancer, and hepatocellular carcinoma, 1 each. Most studies were conducted at a single institution and emphasized feasibility, toxicity, local control, progression-free survival, or overall survival.

**Conclusions:**

This expert-verified inventory shows that CIRT studies open to enrollment remain concentrated by center, disease site, and phase. Future progress may depend more on shared outcome definitions, transparent biological dose reporting, and registry-enabled or multicenter comparative designs than on additional isolated single-institution studies.

## Introduction

A pivotal randomized trial in unresectable salivary gland tumors provided early clinical evidence that fast neutron therapy (FNT), a modality with high linear energy transfer (LET), could improve locoregional control relative to photon therapy.^1^, ^2^ The trial closed early after an interim analysis showed an efficacy difference, but wider adoption of FNT was limited by severe toxicity in some settings and by dosimetric and logistical challenges.^3^

Carbon ion radiotherapy (CIRT) has emerged globally as a promising treatment modality, combining the biological advantages of densely ionizing radiation with the physical dose distribution of charged particles.^4^ These properties make carbon ions especially appealing for radioresistant or hypoxic tumors, rare tumors with limited treatment alternatives, recurrent disease requiring re-irradiation, and radiation-induced tumors, as summarized by the proposed "4Rs" framework for hadrontherapy patient selection.^5^ CIRT is also attractive when organ-at-risk constraints limit dose escalation using low-LET approaches. The biological rationale for CIRT is strongest in disease settings in which local control is limited by tumor biology rather than target geometry and physical dose escalation alone.^6^

CIRT is now established throughout Asia and several European countries, though access remains geographically limited in many Western regions.^4^ As of May 2026, PTCOG listed 17 operational carbon-ion treatment facilities across 7 countries,^7^ and provisional statistics documented 57 500 patients treated with carbon ions through the end of 2023.^8^ Despite its promise, there remains a substantial gap in high-level clinical trial data, in part because treatment is concentrated at a relatively small number of institutions and many of the clinical settings of greatest interest are uncommon and biologically heterogeneous.^6^ Similar to the proton therapy experience, the field has a substantial body of high-quality institutional series and prospective studies but comparatively little mature, practice-changing evidence.^9^

With North America's first clinical carbon-ion therapy program expected to begin treatment in early 2028, the present moment represents an important opportunity to define the clinical questions, trial structures, and reporting standards that will shape early North American experience.^4^ In this context, this expert-verified narrative review provides a structured inventory of CIRT clinical studies open to enrollment worldwide, identifies major gaps in the evidence base, and highlights opportunities for future consensus-building across the international heavy-ion community.

## Materials and methods

This Particle Therapy Cooperative Group (PTCOG)-sanctioned project was conducted as an expert-verified narrative review and inventory within the CIRT Guidelines Subcommittee. Participating centers spanned Japan, China, Taiwan, and South Korea in Asia; Germany, Italy, Austria, and France in Europe; and the United States in North America. Center representatives from the National Institutes for Quantum Science and Technology (QST), Japan; Gunma University Heavy Ion Medical Center, Japan; Shanghai Proton and Heavy Ion Center (SPHIC), China; Institute of Modern Physics, Chinese Academy of Sciences and Lanzhou Heavy Ion Hospital, China; Taipei Veterans General Hospital, Taiwan; Yonsei Cancer Center, Yonsei University Health System, South Korea; Marburg Ion-Beam Therapy Center (MIT), University Hospital Giessen and Marburg (UKGM), Germany; Heidelberg Ion-Beam Therapy Center (HIT), Heidelberg University Hospital, Germany; CNAO National Center for Oncological Hadrontherapy, Italy; MedAustron Ion Therapy Center, Austria; and Grenoble Alpes University, France. Representatives were contacted through the PTCOG Heavy Ion Subcommittee to provide direct input and verification of clinical study activity.

To identify studies open to new enrollment, we queried ClinicalTrials.gov and the Japan Registry of Clinical Trials (jRCT) between December 2025 and February 2026. Registry searches used the term "CIRT" and were limited to English-language records. We included interventional CIRT trials and prospective registries open to new enrollment. Studies not open to new enrollment, including those listed as "Active, not recruiting" or those in treatment, follow-up, or data analysis phases, were excluded, as were retrospective cohorts, proton-only studies, and preclinical work. For each eligible study, data were abstracted on disease site, trial phase, study design (single-arm versus randomized), comparator arm when applicable, and primary endpoint. Because several centers use combined phase designations such as phase I/II or Ib/II, original phase designations were retained in Supplementary Table S1; for summary reporting in Figure 1 and the Results, combined-phase studies were classified according to the highest phase (phase II).

**Figure 1:**
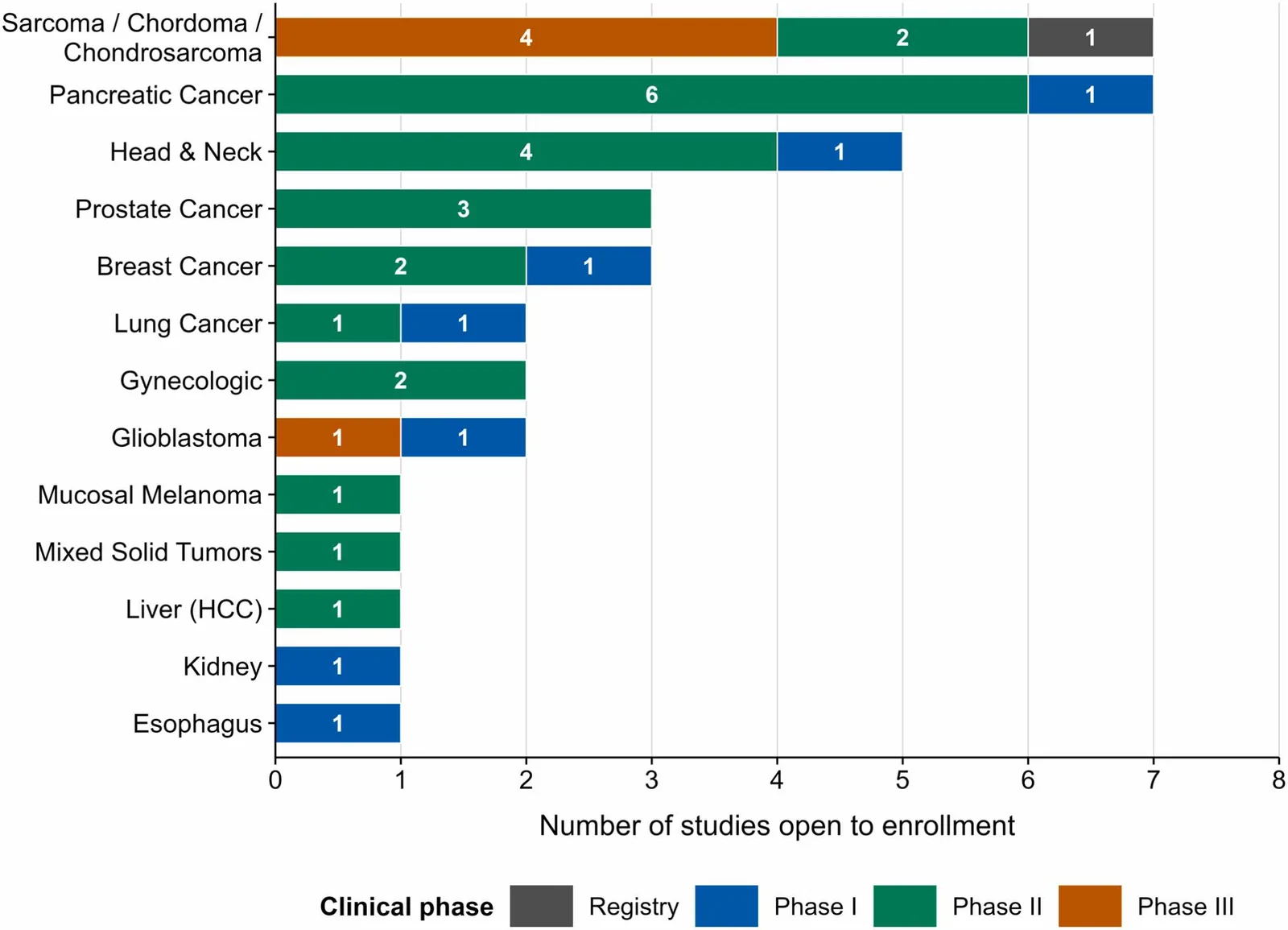
Carbon ion radiotherapy studies open to enrollment by disease site and phase.

Trial listings were then cross-referenced with information provided directly by participating centers and confirmed by members of the PTCOG subcommittee. Representatives from these centers, listed as coauthors on this manuscript, reviewed the trial inventory for accuracy and completeness. These steps were intended to improve completeness and reduce misclassification in a rapidly changing, multinational trial environment.

This review should therefore be interpreted as an expert-verified narrative review and inventory of global CIRT clinical studies open to enrollment rather than as a formal systematic or scoping review. The collaborative framing reflects the purpose of the project: to identify shared clinical-trial priorities, highlight gaps in the current evidence base, and support future multicenter coordination.

For simplicity of reporting, each study was assigned to a single disease-site category to avoid double counting. Pancreatic protocols were grouped regardless of resectability; sarcoma-related tumors included soft tissue sarcoma, chordoma, and chondrosarcoma; nasopharyngeal carcinoma and adenoid cystic carcinoma were grouped under head and neck disease; and mixed-histology protocols were denoted as mixed solid tumors rather than reallocated to one organ-specific category. The primary areas of interest were descriptive: study counts by disease site and phase; institutional and consortium distribution; and the major endpoints used across the portfolio.

## Results

As shown in Figure 1, Figure 2, 36 studies were identified: 35 interventional protocols and 1 registry-based comparative-effectiveness study. When combined phase I/II and Ib/II protocols were classified according to the highest phase, the cohort included 7 phase I studies, 23 phase II studies, 5 phase III studies, and 1 registry study. The 23 studies summarized as phase II comprised 18 pure phase II, 4 phase I/II, and 1 phase Ib/II protocols. Disease-site distribution was led by pancreatic cancer and sarcoma-related tumors, with 7 studies each, followed by head and neck disease with 5 studies, prostate and breast cancer with 3 studies each, and smaller exploratory groups in glioblastoma, lung, gynecologic malignancies, mucosal melanoma, mixed solid tumors, kidney cancer, esophageal cancer, and hepatocellular carcinoma (HCC). Most studies were single-arm, conducted at a single institution, or both; only a minority incorporated randomized or registry-based comparative designs. Original phase designations and the detailed trial inventory are provided in Supplementary Table S1.

**Figure 2:**
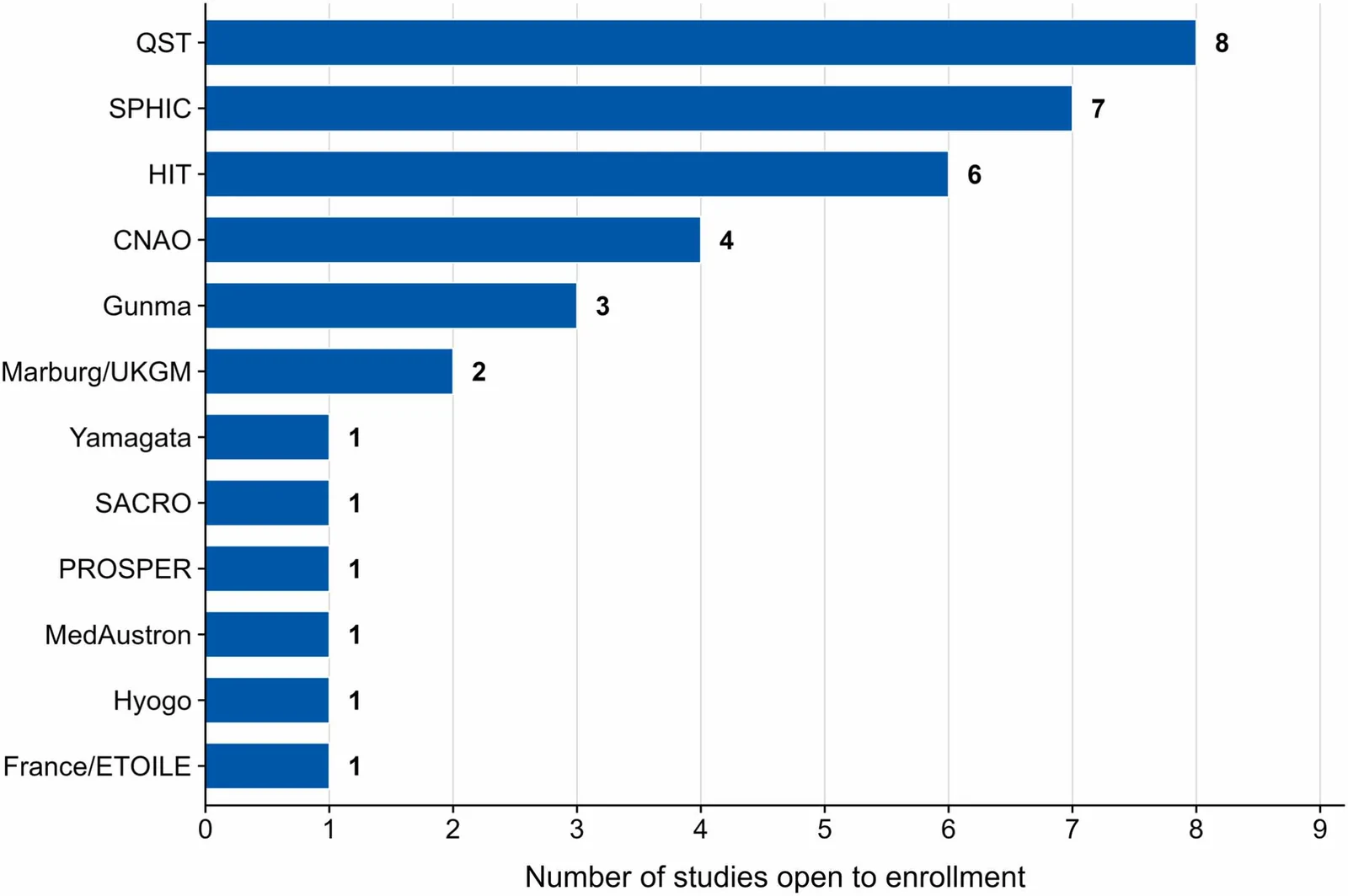
Carbon ion radiotherapy studies open to enrollment by institution or consortium.

Figure 2 illustrates the institutional distribution of the included studies, underscoring the geographic concentration of the portfolio, with most studies open at a small number of high-volume academic centers. The Lyon-sponsored Randomized Carbon Ions vs Standard Radiotherapy for Radioresistant Tumors (ETOILE) trial was labeled France/ETOILE to reflect the transnational nature of that randomized study.^10^, ^11^ Interventional study activity spans Japan, China, Germany, Italy, Austria, and France, while the PROSPER registry provides an open North American comparative-effectiveness platform.^12^

Across the represented trials, the most frequent primary endpoints were safety, feasibility, local control, progression-free survival, overall survival, and disease-specific control metrics such as biochemical relapse-free survival in prostate cancer. Quality-of-life and patient-reported outcomes were included in selected studies, particularly within registry-based and multicenter platforms, but they were not uniformly incorporated across disease groups.

## Discussion

Given the limited number of carbon ion centers worldwide and the resulting constraints on trial access, we performed an international assessment of CIRT clinical studies open to enrollment through the PTCOG CIRT Guidelines Subcommittee. Several themes emerge. First, the study landscape remains concentrated within high-volume academic centers. This concentration reflects the complexity of CIRT, which requires highly specialized multidisciplinary teams able to integrate disease-specific clinical perspectives, radiobiological knowledge, and advanced treatment-planning expertise. Second, the investigated indications cluster around radioresistant biology, proximity to critical anatomy, or clinical situations in which low-LET dose escalation is constrained by organ-at-risk tolerance. Third, prospective comparative evidence remains less mature than the cumulative clinical experience might suggest.^4^, ^6^, ^13^

Pancreatic cancer is one of the 2 most frequently studied disease sites in the current dataset, accounting for nearly 20% of included studies. The rationale is well founded, but the therapeutic and technical challenges are substantial: pancreatic tumors are often unresectable or borderline resectable; local failure can influence outcomes; adjacent gastrointestinal organs, respiratory motion, and changes in bowel filling or peristalsis constrain dose escalation and may challenge range robustness and reproducible dose delivery; and systemic failure remains a major competing risk.^14^, ^15^ In a 2025 phase II study from SPHIC, 49 patients with locally advanced pancreatic carcinoma treated with CIRT plus chemotherapy had a median overall survival of 24.1 months from diagnosis and no grade 3 or higher acute or late toxicity, although the prespecified primary endpoint was not met.^15^ Initial Taiwanese experience with risk-adapted dose-escalated CIRT likewise demonstrated high early local control with low toxicity, but follow-up was short, and systemic failure remained common.^14^ Contemporary photon benchmarks have also evolved. A multicenter phase II study of stereotactic MR-guided on-table adaptive radiation therapy (SMART) reported 2-year overall survival of 53.6% from diagnosis and 2-year local control of 77.7% after 50 Gy/5 treatments in selected patients who had received induction chemotherapy.^16^ NRG-GI011 is now evaluating dose-escalated radiotherapy after induction chemotherapy in a phase III trial.^17^ Future pancreatic CIRT studies should therefore compare against contemporary ablative adaptive radiotherapy rather than conventional chemoradiation alone and should prospectively address motion management, range and robustness evaluation, gastrointestinal dose constraints, systemic therapy integration, and clinically meaningful comparative endpoints.

Sarcoma-related tumors, including soft tissue sarcoma, chordoma, and chondrosarcoma, represent another major area of active investigation. This group contains some of the field's strongest comparative and multinational efforts. The Sacral Chordoma Carbon Ion Radiotherapy (SACRO) study, the ETOILE randomized platform, and the Prospective Registry for Patients Treated with Particle Therapy (PROSPER) align well with the rationale for CIRT: local control matters, surgery may be morbid or incomplete, and radiotherapy often must be delivered near critical structures.^10^, ^11^, ^12^, ^18^ Importantly, the randomized phase II Ion Beam Therapy in Sacrococcygeal Chordoma (ISAC) trial enrolled 82 patients with sacrococcygeal chordoma and reported 2- and 4-year overall survival rates of 96% and 81%, respectively, and corresponding local progression-free survival rates of 84% and 70%; no statistically significant difference was observed between proton and carbon ion therapy within that hypofractionated regimen.^19^ Because ISAC used the local effect model I (LEM-I) for treatment planning, cross-study comparisons with microdosimetric kinetic model (MKM)-based series should be interpreted cautiously. These data underscore the importance of well-designed comparative studies and transparent reporting of physical dose and biological modeling across centers. Continued recruitment of SACRO, ETOILE, and PROSPER reinforces sarcoma-related disease as one of the clearest opportunities for multinational evidence generation, especially when CIRT is being compared with other potentially definitive treatment options.^10^, ^11^, ^12^, ^18^

Head and neck disease remains one of the most biologically compelling but methodologically heterogeneous focus areas for CIRT. The included trials in this group span adenoid cystic carcinoma, nasopharyngeal carcinoma, other uncommon non-squamous malignancies, and recurrent or previously irradiated disease. More broadly, particle therapy has shown potential clinical advantages in selected head and neck settings, including oropharyngeal and sinonasal tumors.^20^, ^21^ Earlier randomized data with FNT also suggested that high-LET therapy can be clinically meaningful for these cancers.^22^

Within that context, CIRT is attractive because it may preserve the dosimetric conformality needed in head and neck cancer while adding biological potency. A 2024 systematic review and meta-analysis of charged-particle therapy for head and neck adenoid cystic carcinoma included 14 studies and 1297 patients and reported pooled 5-year overall survival of 77%, 5-year local control of 73%, and grade 3 or higher late toxicity of 8%.^23^ A subsequent 2025 systematic review included 44 particle-therapy studies, of which 41 were retrospective and 3 were prospective, and likewise reported favorable modality-specific control with acceptable toxicity.^24^ These outcomes are encouraging in a rare and often locally recalcitrant disease, but cross-study heterogeneity, selection bias, and the limited prospective evidence make comparative interpretation challenging. The active trial portfolio mirrors these same challenges: there is sustained interest in CIRT-based definitive therapy and re-irradiation, but the evidence base remains highly center-dependent.

By comparison, prostate cancer is represented by 3 included phase II studies and published single-center series report favorable biochemical control with low late toxicity. A 2024 cohort of 253 patients treated with scanning CIRT to 51.6 Gy-Relative Biological Effectiveness (RBE) in 12 fractions over 3 weeks reported 5-year overall survival of 97.5% and biochemical relapse-free survival of 93.3%, with 5-year cumulative grade 2 or greater late genitourinary and gastrointestinal toxicity rates of 7.4% and 1.2%, respectively.^25^ These outcomes are encouraging and support further evaluation of hypofractionated CIRT approaches. At the same time, prostate cancer may not be the setting in which carbon provides the clearest biological advantage over other highly conformal modalities such as proton therapy or stereotactic ablative radiotherapy. Future prostate studies may therefore be most informative when they focus on biologically selected populations, nodal or very-high-risk disease, or patient-reported outcomes that are difficult to capture in historical series. The same principle applies to breast cancer, where CIRT studies should be linked to a defined unmet need, such as selected re-irradiation or organ-preservation scenarios, rather than general substitution for established local therapies.

Other disease groups represented in the included portfolio include breast, lung, gynecologic malignancies, glioblastoma, HCC, kidney cancer, esophageal cancer, mucosal melanoma, and mixed solid tumors. These studies demonstrate the breadth of current clinical activation, but they should still be interpreted as exploratory. High-grade glioma and other radioresistant tumors near critical structures may merit coordinated study when the biological rationale, normal-tissue constraints, and comparative question are explicit. Together, these studies broaden the potential applications of heavy-ion therapy across organ preservation, focal ablative treatment, re-irradiation, and combination therapy settings. At the same time, the expansion of indications carries a risk of fragmentation if trials are not anchored by common reporting standards and realistic multinational accrual strategies.

A fundamental principle in the development of CIRT is that its application should be driven by unmet clinical needs rather than by technological availability alone. Collaboration with the broader oncology community is therefore essential to identify relevant unmet needs, determine whether CIRT has the potential to address them, and define the clinical scenarios in which its added value can be realistically demonstrated. Priority domains include organ-preserving strategies that may avoid disabling surgery, radioresistant or unresectable tumors for which local control remains inadequate, and translational studies of the tumor microenvironment and immunogenic effects, particularly in combination with systemic therapies such as immunotherapy. Comparative trials should test a clinically and biologically meaningful CIRT strategy rather than simply match nominal RBE-weighted doses across modalities, because model-dependent dose equivalence may obscure the hypothesized advantage of CIRT.^26^, ^27^ Consequently, prospective CIRT trials should be aligned with meaningful clinical endpoints and the specific unmet need that the technology is intended to address. The absence of pediatric studies in the current inventory also supports engagement with pediatric oncology cooperative groups to identify rare high-risk histologies, define age-appropriate endpoints, and avoid simple extrapolation of adult protocols.

A major issue observed across studies was endpoint heterogeneity. Because much of the included portfolio is phase I or phase II, it is unsurprising that many trials prioritize maximum tolerated dose, safety, or feasibility. Others use local control, local progression-free survival, progression-free survival, overall survival, biochemical relapse-free survival, wound-healing outcomes, quality of life, or other patient-reported outcomes. Some variation is inevitable and appropriate because disease biology and clinical questions differ by site. Nevertheless, the current level of heterogeneity limits pooled interpretation. Future CIRT trials would be strengthened by disease-specific core outcome sets that include one principal disease-control endpoint together with harmonized toxicity reporting and a consistent patient-reported outcome component.

An additional issue that is intrinsic to CIRT is the reporting of biological dose assumptions. Because CIRT data interpretation depends partly on enhanced biological effectiveness rather than geometry alone, RBE model selection should not remain a technical afterthought. LEM and MKM approaches can yield materially different RBE-weighted dose distributions even when the underlying physical dose distributions appear similar, and translation between models requires caution.^26^, ^27^ Ongoing efforts also extend beyond physical dose optimization to LET-optimized planning.^28^, ^29^, ^30^ For future clinical trial manuscripts, reporting of the RBE model, prescription basis, treatment planning system, LET-related quantities, model-translation strategy, and standardized acute and late toxicity outcomes should become standard rather than optional.

Accordingly, the current trial portfolio suggests a practical international agenda. Immediate opportunities include prioritizing collaborative accrual in pancreatic cancer and sarcoma, establishing disease-specific common outcome sets in head and neck and pelvic disease, and leveraging registry-based comparative-effectiveness designs when randomization is unlikely to be feasible. PROSPER provides one model for pragmatic multinational evidence generation, while SACRO and ETOILE provide disease-focused structures for cross-center participation.^10^, ^11^, ^12^, ^18^ Future North American carbon-ion capacity could make similar designs more realistic for other rare diseases.

Successful multinational CIRT trials will require more than shared protocols. Participation can be streamlined through a central coordinating office, standardized contracts and data elements, site credentialing, benchmark cases, common contouring and planning atlases, digital plan transfer, rapid central review, and prospective individual-case radiotherapy quality assurance. These approaches are established in cooperative-group radiotherapy trials and are especially important for CIRT because centers differ in beam delivery, dose notation, treatment-planning systems, RBE models, and clinical experience.^31^, ^32^ Published operational quality-assurance details for individual CIRT platforms remain limited, but SACRO, ETOILE, and PROSPER illustrate the value of disease-focused, multi-institutional outcome collection.

As CIRT programs expand, referral pathways from non-CIRT centers will also influence trial accrual and appropriate patient selection. Practical strategies include disease-specific referral criteria, rapid virtual multidisciplinary review, standardized imaging and pathology checklists, financial and logistical navigation, and timely communication with referring teams. Appropriate trial candidates should have a defined unmet need, protocol eligibility, adequate performance status, and a plausible biological or dosimetric rationale for CIRT. Patients with uncontrolled disease progression, inability to safely meet motion-management or organ-at-risk constraints, or no credible incremental value over accessible standard therapy may be less suitable for referral. These principles should be applied through personalized multidisciplinary review rather than as stringent exclusions.

As a radiotherapy innovation, CIRT can reasonably be considered a radiation-centered intervention within the ESTRO Value-Based Radiation Oncology framework because its principal innovation lies in radiation-specific biological and dosimetric properties.^33^ The level of evidence should be proportional to the claim: early-phase single-arm studies can establish feasibility and safety, whereas claims of added clinical value intended to support implementation should rely on randomized comparative evidence when feasible. In selected settings, pragmatic trials and high-quality prospective observational designs may be appropriate alternatives, and endpoints should extend beyond overall survival to include local control, toxicity, quality of life, and functional outcomes.^34^

Beyond cataloging the included studies, a priority for the field is harmonizing how CIRT trials are reported. Based on the findings of the present review, the manuscript authors developed and propose a minimum reporting framework spanning 6 domains as illustrated in Table 1: trial identity and design; clinical context and eligibility; a common endpoint core anchored by disease-appropriate efficacy, toxicity, and quality-of-life measures; transparent reporting of prescription and achieved target coverage; explicit disclosure of organ-at-risk constraints and any intentional target undercoverage; and standardized reporting of the treatment planning system, RBE model, and LET-related quantities. Such a framework would make current single-institution studies more interpretable, improve cross-center comparability, and create the foundation for prospectively planned pooled analyses and future multicenter trials.

**Table 1:** Author-proposed minimum reporting standards for current and future CIRT clinical studies.

Standard	Reporting elements
Trial identity and design	Registry identifier; center or consortium; country/region; trial phase; accrual status; single-arm versus randomized design; comparator arm when applicable; planned sample size; and data cut-off date for registry-based reports.
Clinical context and eligibility	Histology, anatomic site, stage/classification system used, operable versus unresectable status, prior radiotherapy status, re-irradiation interval, systemic therapy plan, gross tumor volume, nodal status, and key baseline imaging/staging studies.
Endpoint framework	Phase-appropriate primary endpoint plus relevant secondary endpoints: local control or disease-appropriate local efficacy metric; locoregional control when clinically relevant; progression-free, distant metastasis-free, and overall survival as relevant; acute toxicity; late toxicity; and patient-reported outcomes/quality of life. Include assessment schedule, imaging modality, and confirmation method for treatment failure.
Prescription and target coverage	Physical dose and RBE-weighted dose units; fractionation; delivery technique; motion management; adaptive replanning; target structures used for optimization and evaluation; target coverage metrics (e.g., D98, D95, D50, D2, V95); robustness approach; and PTV or evaluation-volume coverage depending on planning paradigm.
OAR constraints and protocol deviations	Organ-specific constraints; relevant metrics such as Dmax, D0.03cc, D1cc, Dmean, or Vx; target-OAR overlap volume; intentional target undercoverage used to respect OAR limits; cumulative dose assumptions in re-irradiation; and protocol deviation or QA classification.
Biological model and pooled-analysis readiness	Treatment planning system and version; beam model; RBE model used for optimization and reporting (e.g., LEM or MKM variant); dose nomenclature; LET metric used and whether LET-informed optimization was performed; model-translation method if used; and minimal common dataset for failures, toxicity, PROs, and follow-up timepoints.

Abbreviations: CIRT, carbon ion radiotherapy; LET, linear energy transfer; LEM, local effect model; MKM, microdosimetric kinetic model; OAR, organ at risk; PRO, patient-reported outcome; PTV, planning target volume; QA, quality assurance; RBE, relative biological effectiveness; TPS, treatment planning system. D98, D95, D50, and D2 denote the dose received by at least 98%, 95%, 50%, and 2% of the evaluated volume, respectively; V95 refers to the volume receiving at least 95% of the prescription dose.

Because this review focused on studies open to enrollment, studies listed as "Active, not recruiting" and those in treatment, follow-up, or data analysis phases were not included. These studies are especially important to near-term evidence generation, and a complementary outcome-focused review will be needed as their results mature.

Limitations include the dynamic nature of clinical trial registries, institutional variation in public reporting, and incomplete public-facing study descriptions. The search was limited to ClinicalTrials.gov and jRCT, used an English-language search term, and included registry status through February 2026; studies registered exclusively in other national registries or opened after the search cutoff may therefore have been missed. The included portfolio also contained no pediatric studies. Direct verification by representatives from major participating centers reduced, but could not eliminate, these limitations. The report should therefore be interpreted as a practical snapshot rather than a permanent census of the CIRT trial landscape, and centers without designated PTCOG representatives may be underrepresented.

## Conclusion

We conducted an international narrative review of CIRT clinical studies open to enrollment and identified 36 studies, including 35 interventional protocols and 1 registry-based study. The included portfolio is concentrated primarily in pancreatic cancer, sarcoma-related tumors, and head and neck disease. The CIRT clinical study portfolio remains concentrated at a small number of centers, and most studies are early phase or nonrandomized. A coordinated international strategy built around shared outcome sets, transparent biological dose reporting, streamlined referral pathways, prospective radiotherapy quality assurance, and multicenter accrual is needed to convert promising institutional experience into practice-changing evidence.

Bars show the number of included registry-based and interventional CIRT studies by disease-site group and clinical phase. Combined phase I/II and Ib/II studies were classified according to the highest phase (phase II) for summary reporting. Numbers within each bar segment indicate the number of studies in that clinical phase. CIRT, carbon ion radiotherapy; HCC, hepatocellular carcinoma.

Bars show the number of included CIRT studies assigned to each institution, registry, or consortium in the study inventory. Numbers at the end of each bar indicate the number of studies. QST, National Institutes for Quantum Science and Technology; HIMAC, Heavy Ion Medical Accelerator in Chiba; SPHIC, Shanghai Proton and Heavy Ion Center; HIT, Heidelberg Ion-Beam Therapy Center; CNAO, Centro Nazionale di Adroterapia Oncologica; MIT, Marburg Ion-Beam Therapy Center; UKGM, University Hospital Giessen and Marburg; ETOILE, France-based transnational randomized CIRT platform; SACRO, Sacral Chordoma Carbon Ion Radiotherapy; PROSPER, Prospective Registry for Patients Treated with Particle Therapy.

## Funding

This research did not receive any specific grant from funding agencies in the public, commercial, or not-for-profit sectors.

## CRediT authorship contribution statement

Adam L. Holtzman: Conceptualization, Methodology, Data curation, Validation, Project administration, Supervision, Visualization, Writing – original draft, Writing – review and editing. Bradford S. Hoppe : Conceptualization, Methodology, Validation, Supervision, Writing – review and editing. Huan Giap, Hitoshi Ishikawa, Tatsuya Ohno, Guoliang Jiang, Qiang Li, Keng-Li Lan, Woong Sub Koom, Sebastian Adeberg, Ester Orlandi, Piero Fossati, Eugen Hug, Jacques Balosso, and Semi Harrabi: Investigation, Resources, Validation, Writing – review and editing. All authors reviewed the final manuscript, verified the accuracy of information contributed from their respective institutions or regions when applicable, approved the final version for submission, and agree to be accountable for the integrity and accuracy of the work.

## Declaration of Generative AI and AI-Assisted Technologies in the Manuscript Preparation Process

During the preparation of this work, the authors used OpenAI ChatGPT (GPT-5.6 Sol Pro) to assist with language editing, manuscript refinement, and refining R code used to generate and format Figure 1, Figure 2. The authors reviewed and edited all AI-assisted content, independently verified the code outputs and resulting figures against the underlying data, and take full responsibility for the content of the publication.

## Declaration of Conflicts of Interest

The authors declare that they have no known competing financial interests or personal relationships that could have appeared to influence the work reported in this paper.

## Footnotes

Appendix A Supplementary data associated with this article can be found in the online version at doi:10.1016/j.ijpt.2026.101968.

## References

[1] Griffin T.W., Pajak T.F., Laramore G.E. (1988). Neutron vs photon irradiation of inoperable salivary gland tumors: results of an RTOG-MRC Cooperative Randomized Study. Int J Radiat Oncol Biol Phys.

[2] Laramore G.E., Krall J.M., Griffin T.W. (1993). Neutron versus photon irradiation for unresectable salivary gland tumors: final report of an RTOG-MRC randomized clinical trial. Int J Radiat Oncol Biol Phys.

[3] Gordon K., Gulidov I., Fatkhudinov T., Koryakin S., Kaprin A. (2022). Fast and furious: fast neutron therapy in cancer treatment. Int J Part Ther.

[4] Liang X., Mohammadi H., Moreno K.C., Beltran C.J., Holtzman A.L. (2025). Heavy ion particle therapy in modern day radiation oncology. Hematol Oncol Clin North Am.

[5] Vischioni B., Barcellini A., Magro G. (2024). Radioresistant, rare, recurrent, and radioinduced: 4Rs of hadrontherapy for patients selections. Int J Part Ther.

[6] Malouff T.D., Mahajan A., Krishnan S., Beltran C., Seneviratne D.S., Trifiletti D.M. (2020). Carbon ion therapy: a modern review of an emerging technology. Front Oncol.

[7] Particle Therapy Co-Operative Group. Facilities in clinical operation. Updated May 2026. Accessed August 10, 2026.

[8] Particle Therapy Co-Operative Group. Particle Therapy Patient Statistics (per end of 2023, provisional). Updated April 2024.

[9] Odei B.C.L., Boothe D., Keole S.R. (2017). A 20-year analysis of clinical trials involving proton beam therapy. Int J Part Ther.

[10] Balosso J., Febvey-Combes O., Iung A. (2022). A randomized controlled phase III study comparing hadrontherapy with carbon ions versus conventional radiotherapy - including photon and proton therapy - for the treatment of radioresistant tumors: the ETOILE trial. BMC Cancer.

[11] ClinicalTrials.gov. Randomized carbon ions vs standard radiotherapy for radioresistant tumors (ETOILE). NCT02838602. Accessed December 13, 2025.

[12] Hoppe B.S., Petersen I.A., Wilke B.K. (2023). Pragmatic, prospective comparative effectiveness trial of carbon ion therapy, surgery, and proton therapy for the management of pelvic sarcomas (soft tissue/bone) involving the bone: the PROSPER study rationale and design. Cancers (Basel).

[13] Jang J.Y., Kim K., Chen M.-F. (2024). A meta-analysis comparing efficacy and safety between proton beam therapy versus carbon ion radiotherapy. Cancer Med.

[14] Lai I.-C., Kang Y.-M., Wu Y.-H. (2026). Carbon ion therapy for pancreatic cancer with risk-adapted dose escalation: initial clinical experience. Radiother Oncol.

[15] Zhang G., Cai X., Cao H. (2025). Prospective phase 2 clinical trial of carbon ion radiation therapy combined with chemotherapy for locally advanced pancreatic carcinoma. Int J Radiat Oncol Biol Phys.

[16] Chuong M.D., Lee P., Low D.A. (2024). Stereotactic MR-guided on-table adaptive radiation therapy (SMART) for borderline resectable and locally advanced pancreatic cancer: a multi-center, open-label phase 2 study. Radiother Oncol.

[17] ClinicalTrials.gov. Testing Higher Dose Radiation Therapy for Locally Advanced Pancreatic Cancer (NRG-GI011/LAP100). NCT06958328. Accessed September 22, 2026.

[18] Fossati P., Radaelli S., Hug E.B. (2026). Sacral chordoma: a randomized and observational study on surgery vs definitive particle-based radiotherapy in primary localized disease (SACRO). Int J Part Ther.

[19] Seidensaal K., Froehlke A., Lentz-Hommertgen A. (2024). Hypofractionated proton and carbon ion beam radiotherapy for sacrococcygeal chordoma (ISAC): an open label, randomized, stratified, phase II trial. Radiother Oncol.

[20] Frank S.J., Busse P.M., Lee J.J. (2026). Proton versus photon radiotherapy for patients with oropharyngeal cancer in the USA: a multicentre, randomised, open-label, non-inferiority phase 3 trial. Lancet.

[21] Patel S.H., Wang Z., Wong W.W. (2014). Charged particle therapy versus photon therapy for paranasal sinus and nasal cavity malignant diseases: a systematic review and meta-analysis. Lancet Oncol.

[22] Maor M.H., Errington R.D., Caplan R.J. (1995). Fast-neutron therapy in advanced head and neck cancer: a collaborative international randomized trial. Int J Radiat Oncol Biol Phys.

[23] Tan M., Chen Y., Du T. (2024). Assessing the impact of charged particle radiotherapy on head and neck adenoid cystic carcinoma: a systematic review and meta-analysis. Technol Cancer Res Treat.

[24] Rohit A., Bates J.E., Frank S.J. (2025). A systematic review of particle-based treatment modalities for adenoid cystic carcinomas of the head and neck. Radiother Oncol.

[25] Takakusagi Y., Koge H., Kano K. (2024). Five-year clinical outcomes of scanning carbon-ion radiotherapy for prostate cancer. PLoS One.

[26] Góra J., Grosshagauer S., Fossati P. (2024). The sensitivity of radiobiological models in carbon ion radiotherapy (CIRT) and its consequences on the clinical treatment plan: differences between LEM and MKM models. J Appl Clin Med Phys.

[27] Karger C.P., Peschke P. (2018). RBE and related modeling in carbon-ion therapy. Phys Med Biol.

[28] Parrella G., Magro G., Chalaszczyk A. (2024). Balancing benefits and limitations of linear energy transfer optimization in carbon ion radiotherapy for large sacral chordomas. Phys Imaging Radiat Oncol.

[29] Baltazar F., Longarino F.K., Stengl C. (2025). Investigating LETd optimization strategies in carbon ion radiotherapy for pancreatic cancer: a dosimetric study using an anthropomorphic phantom. Med Phys.

[30] Koto M., Ikawa H., Inaniwa T. (2024). Dose-averaged LET optimized carbon-ion radiotherapy for head and neck cancers. Radiother Oncol.

[31] Weber D.C., Poortmans P.M.P., Hurkmans C.W., Aird E., Gulyban A., Fairchild A. (2011). Quality assurance for prospective EORTC radiation oncology trials: the challenges of advanced technology in a multicenter international setting. Radiother Oncol.

[32] Fairchild A., Bar-Deroma R., Collette L. (2012). Development of clinical trial protocols involving advanced radiation therapy techniques: the European Organisation for Research and Treatment of Cancer Radiation Oncology Group approach. Eur J Cancer.

[33] Vandemaele M., Blanchard P., Borràs J.M. (2025). Building an appraisal framework for radiotherapy innovations in a value-based context: the ESTRO-value-based radiation oncology categorisation system. Radiother Oncol.

[34] Vandemaele M., Leech M., Aznar M. (2026). An international modified Delphi study to prioritise levels of evidence and outcomes to appraise radiotherapy innovation in the ESTRO Value-Based Radiation Oncology framework. Lancet Oncol.

