# Supplementary material for: Global Landscape of Clinical Studies Open to Enrollment in Carbon Ion Radiotherapy: A PTCOG Review
Source: Int J Part Ther. 2026 Oct 6;22:101968. doi: 10.1016/j.ijpt.2026.101968 (PMC13675797; doi:10.1016/j.ijpt.2026.101968)
Supplement: Supplementary file 1 [file mmc1.docx]

**Supplementary Table S1. Open and recruiting carbon ion radiotherapy clinical studies included in the review.**

| **Disease-site group** | **Institution/consortium** | **Clinical phase** | **Disease site/focus** | **Registry ID** | **Primary/key endpoint(s)** |
| --- | --- | --- | --- | --- | --- |
| Pancreatic Cancer | Hyogo | I | Pancreas (locally advanced) | — | Maximum tolerated dose |
| Pancreatic Cancer | Gunma | I/II | Pancreas (borderline resectable/unresectable) | jRCTs031230335 | Resection rate; postoperative complications |
| Pancreatic Cancer | Yamagata | I/II | Pancreas (locally advanced) | UMIN000049457 | Morbidity |
| Pancreatic Cancer | MedAustron | II | Pancreas (locally advanced pancreatic cancer) | NCT05191940 | 2-year locoregional control |
| Pancreatic Cancer | QST | II | Pancreas (locally advanced) | jRCTs032210292 | 2-year overall survival |
| Pancreatic Cancer | SPHIC | II | Pancreatic cancer (non-resectable) | NCT05424159 | 2-year local progression rate; 2-year overall survival; acute and late toxicity |
| Pancreatic Cancer | SPHIC | II | Pancreatic carcinoma | NCT05830019 | 2-year local progression-free survival; 2-year overall survival; 2-year progression-free survival; adverse events |
| Sarcoma / Chordoma / Chondrosarcoma | HIT | II | Extremity soft tissue sarcoma | NCT04946357 | Wound-healing-free treatment completion |
| Sarcoma / Chordoma / Chondrosarcoma | HIT | II | Retroperitoneal soft tissue sarcoma | NCT04219202 | Grade 3-5 toxicity; feasibility/practicability |
| Sarcoma / Chordoma / Chondrosarcoma | HIT | III | Chondrosarcoma (skull base) | NCT01182753 | Local-progression-free survival |
| Sarcoma / Chordoma / Chondrosarcoma | HIT | III | Chordoma (skull base) | NCT01182779 | Local-progression-free survival |
| Sarcoma / Chordoma / Chondrosarcoma | France/ETOILE | III | Chordoma, adenoid cystic carcinoma, sarcoma | NCT02838602 | Progression-free survival; local progression-free survival; metastasis-free survival; overall survival; toxicity; quality of life |
| Sarcoma / Chordoma / Chondrosarcoma | SACRO consortium | III | Sacral chordoma (multisite SACRO) | NCT02986516 | Relapse-free survival; overall survival; survival post-progression; local and distant relapse; best response; quality of life |
| Sarcoma / Chordoma / Chondrosarcoma | PROSPER registry | Registry | Pelvic sarcomas (multisite PROSPER) | NCT05033288 | Comparative-effectiveness endpoints |
| Head & Neck | QST | I | Head and neck tumors | jRCTs032240451 | Safety |
| Head & Neck | CNAO | II | Head and neck adenoid cystic carcinoma | NCT05733910 | Acute/subacute toxicity; local control; predictive toxicity modeling |
| Head & Neck | HIT | II | Adenoid cystic carcinoma | NCT04214366 | Freedom from locoregional progression |
| Head & Neck | HIT | II | Recurrent/progressive head and neck cancer | NCT04185974 | Acute/subacute grade 3 or higher toxicity at 6 months |
| Head & Neck | SPHIC | II | Nasopharyngeal carcinoma | NCT04143984 | Progression-free survival; overall survival; local/regional/distant control; adverse events |
| Prostate Cancer | QST | II | Prostate cancer (intermediate risk) | jRCT1032220391 | Biochemical relapse-free survival |
| Prostate Cancer | SPHIC | II | Prostate cancer | NCT05010343 | Acute/late toxicity; biochemical failure-free survival; overall survival; progression-free survival |
| Prostate Cancer | SPHIC | II | Prostate cancer with lymph-node metastases | NCT05106699 | Biochemical relapse-free survival; progression-free survival; overall survival; metastasis-free survival; acute/late toxicity |
| Breast Cancer | SPHIC | I | Breast cancer (TNBC, HER2+) | NCT05692661 | Acute toxicity; local recurrence; cosmetic outcome |
| Breast Cancer | QST | I/II | Breast cancer (T1N0M0) | jRCTs032200066 | Efficacy and safety |
| Breast Cancer | QST | I/II | Breast cancer (T1N0M0) | jRCTs032180154 | Efficacy and safety |
| Glioblastoma | Marburg | I | Glioblastoma (WHO grade IV) | — | Maximum tolerated dose (NCI-CTCAE v5) |
| Glioblastoma | Marburg | III | Recurrent glioblastoma | DRKS00025069 | Overall survival |
| Lung Cancer | Gunma | I | Non-small cell lung cancer | jRCTs031190126 | Maximum tolerated dose of S-1 |
| Lung Cancer | SPHIC | II | Non-small cell lung cancer | NCT05613452 | Progression-free survival; local control; overall survival; adverse events |
| Gynecologic | CNAO | II | Gynecologic malignancies | NCT05457595 | Local control; response; overall survival; toxicity; progression-free survival |
| Gynecologic | Gunma | II | Cervical cancer | NCT06805864; jRCTs031240651 | 2-year progression-free survival; overall survival; local control; complete response; toxicity |
| Mucosal Melanoma | CNAO | II | Mucosal melanoma (lower genital tract) | NCT05478876 | 2-year progression-free survival; 3-year overall survival; toxicity; local response; quality of life |
| Mixed Solid Tumors | CNAO | II | Multiple solid tumors | NCT05229614 | Objective response rate; toxicity |
| Kidney | QST | I | Kidney cancer (T1-3N0M0) | — | Safety |
| Esophagus | QST | I | Esophageal cancer (T1N0M0) | jRCT1032250012 | Safety |
| Liver (HCC) | QST | Ib/II | Hepatocellular carcinoma (>4 cm) | jRCT2031240284 | Safety |

Abbreviations: CIRT, carbon ion radiotherapy; QST, National Institutes for Quantum Science and Technology; HIMAC, Heavy Ion Medical Accelerator in Chiba; SPHIC, Shanghai Proton and Heavy Ion Center; HIT, Heidelberg Ion-Beam Therapy Center; CNAO, Centro Nazionale di Adroterapia Oncologica; UKGM, University Hospital Giessen and Marburg; ETOILE, Randomized Carbon Ions vs Standard Radiotherapy for Radioresistant Tumors; SACRO, Sacral Chordoma Carbon Ion Radiotherapy; PROSPER, Prospective Registry for Patients Treated with Particle Therapy; HCC, hepatocellular carcinoma; TNBC, triple-negative breast cancer; HER2, human epidermal growth factor receptor 2; WHO, World Health Organization; NCI-CTCAE, National Cancer Institute Common Terminology Criteria for Adverse Events; PFS, progression-free survival; OS, overall survival; QoL, quality of life. Registry prefixes: NCT, ClinicalTrials.gov; jRCT, Japan Registry of Clinical Trials; UMIN, University Hospital Medical Information Network Clinical Trials Registry; DRKS, German Clinical Trials Register.
